# Collecting wood core samples from Macassar ebony (*Diospyros celebica* Bakh.) for multi-purpose analysis using pickering punch

**DOI:** 10.1016/j.mex.2022.101728

**Published:** 2022-05-14

**Authors:** Iskandar Zulkarnaen Siregar, Muhammad Majiidu, Fifi Gus Dwiyanti, Essy Harnelly, Ratih Damayanti, Lina Karlinasari, Mohamad Rafi, Dewi Anggraini Septaningsih, Meaghan Parker-Forney

**Affiliations:** a Advanced Research Laboratory, IPB University, Bogor, Indonesia; b Department of Silviculture, Faculty of Forestry and Environment, IPB University, Bogor, Indonesia; c Department of Biology, Faculty of Mathematics and Natural Science, Syiah Kuala University, Banda Aceh, Indonesia; d Research Center for Biomass and Bioproducts, National Research and Innovation Agency (BRIN), Cibinong, Bogor, Indonesia; e Department of Forest Product, Faculty of Forestry and Environment, IPB University, Bogor, Indonesia; f Department of Chemistry, Faculty of Mathematics and Natural Science, IPB University, Bogor, Indonesia; g World Resources Institute, Washington DC, United State

**Keywords:** *Pickering punch*, Ebony, Wood Core

## Abstract

Sample collection activities for a study of population genetics across the natural distribution of targeted tree species require a lot of resources, mainly if repeated field visits are necessary. Conventionally, population genetic studies use good sample material like leaves. In addition, cambium or small pieces of fresh wood can be used to replace leaf samples. Currently, restrictions from the permit regulation have caused only a limited number of samples that can be collected. Therefore, efficient use of samples must be designed to maximize their uses for research. Due to the small amount of successfully sampled materials, hence there are limitations to extend their uses for other analyses and are often sufficient only for genetic analysis. Therefore, innovation in sampling methods using pickering punch (https://www.agroisolab.com/pickering-punch) to collect ebony wood cores in this study is required to cover multi-analyses not only limited to genetics but also for other analyses such as isotopes, near-infrared spectroscopy (NIRs), anatomy, and chemical compounds.

• Pickering punch is recommended for efficient wood core sample collection from ebony standing trees.
• 323 wood core samples were successfully collected from 16 natural populations across Celebes (Sulawesi).
• Multi-analyses studies on sampled wood cores are possible for ebony wood identification (e.g., species and origin/provenance).

## Graphical abstract

**Figure:**
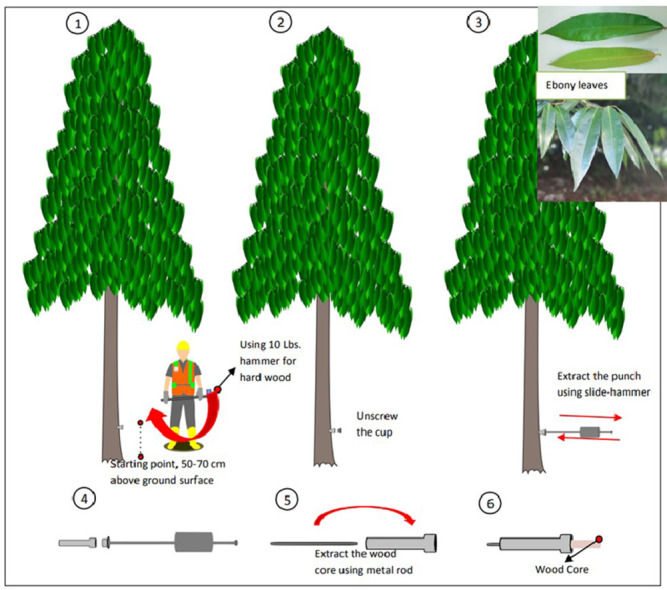

Specifications table

**Table:** 

Subject Area:	Biochemistry, Genetics and Molecular Biology
More specific subject area:	Forest Genetics, Wood Anatomy, Near Infra-Red, Isotopes, and Metabolomics
Method name:	**Technology-based s**ampling for multi-analyses
Name and reference of original method:	Not applicable
Resource availability:	https://www.agroisolab.com/pickering-punch

## Method details

### Wood cores extraction

**Fig. 1:**
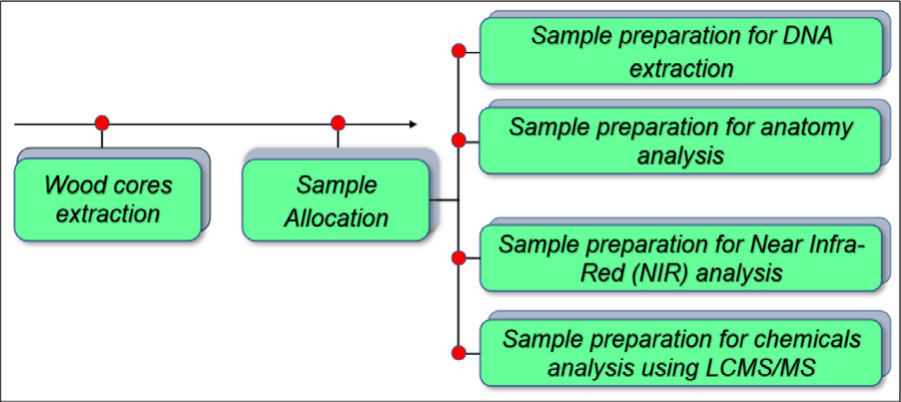
Flowchart of the innovative collection of wood core samples from ebony.

**Fig. 2:**
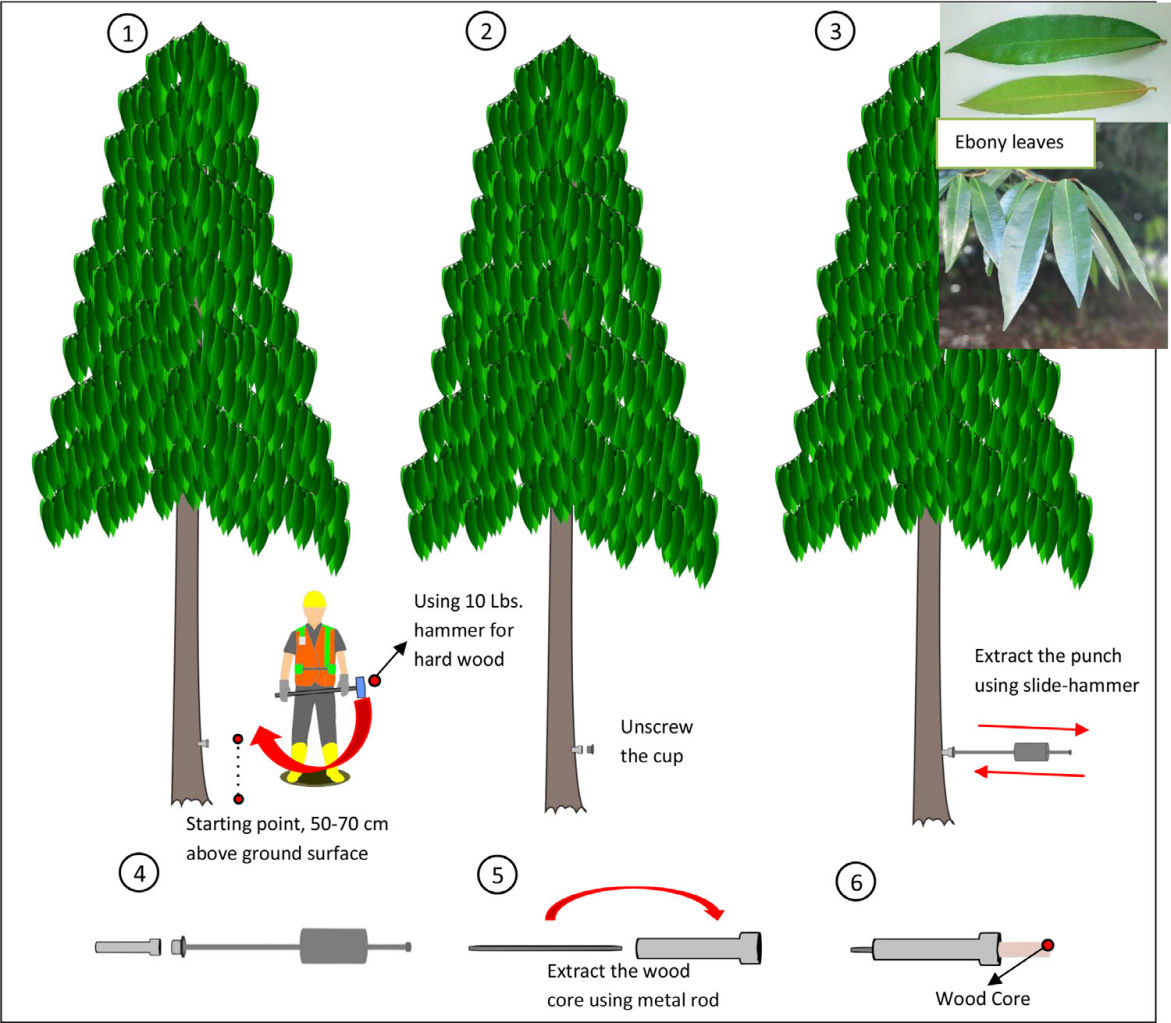
Steps on wood core extraction.

**Fig. 3:**
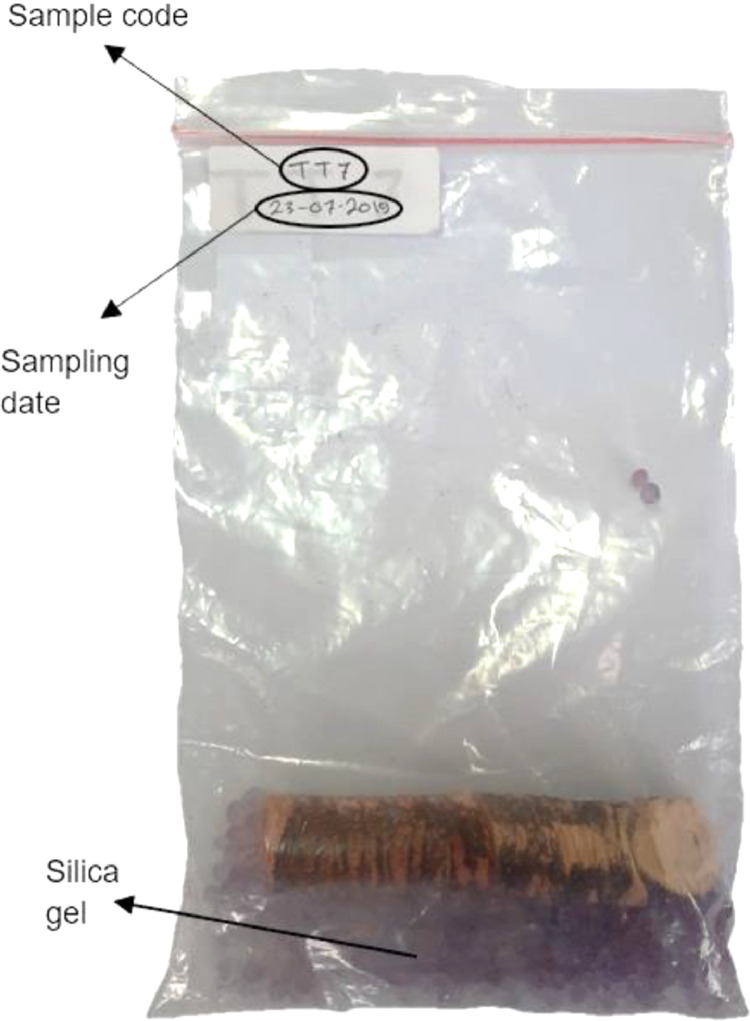
Wood core in a plastic zip lock bag (12 cm x 20 cm).

The workflow for innovative wood core collection is presented in Fig. 1. The equipment and materials required for the extraction of the wood core are 5 and 10 lbs of sledge hammer (depending on the hardness of the wood in which Macassar ebony wood density is around 0.83 kg *m* ^−^ ^3^ [1]), punch, end-cap, slide-hammer, metal rod, silica gel, and petroleum jelly. The starting point on the tree for wood core extraction is about 50–70 cm above the ground. The wood core extraction follows the manufacture protocol by Agroisolab North Yorkshire UK as presented in Fig. 2. Take the end-cap and screw it into the punch and tighten until no thread is exposed. Place the punch on the tree on the right or left side of the tree (left side for left-handed),and then start punching with little taps using 5 lbs hammer. Slightly knock the punch into the tree with 90° angle, and then use 10 lbs of hammer for deeper insertion. Swing the hammer steadily until the punch reach 12 cm depth. Unscrew the end-cap and fasten the slide-hammer, then tighten until no thread is exposed. Slide the weight of the hammer back against the stop point to extract the punch. In addition, remove the slide-hammer and remove the wood core with a metal rod. Place the wood core in the plastic zipper with the silica gel for storage [2] Fig. 3. Cover the extraction hole to shield the tree from insects and fungi with petroleum jelly. The minimum sample size for each population is 20 individuals [3], and each individual has a duplicate of the wood core. In total, 323 ebony wood core samples from 16 populations across Celebes were successfully collected using this method in the present study.

### Sample allocation

**Fig. 4:**
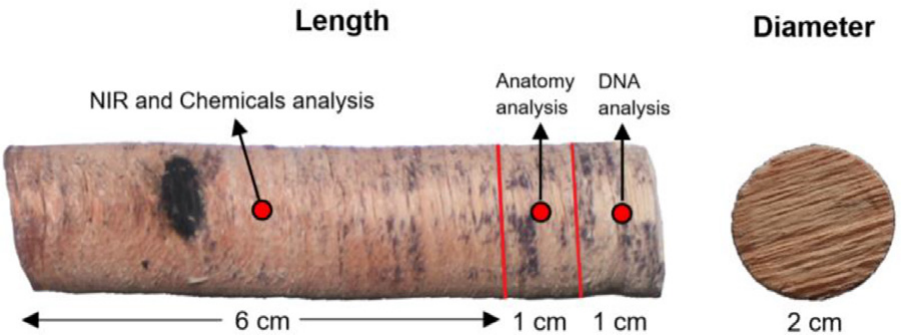
Wood core extracted using the pickering punch.

Wood core samples may be used for several analyses, such as genetics andgenomics [4], anatomy, near infra-red (NIRs) [5], isotopes, and chemicals. The average length of the wood core from the 323 samples was 8 cm with a diameter of 2 cm, from which 1 cm of the wood core is allocated for DNA analysis, another 1 cm is used for anatomy analysis, while the remaining 6 cm is used for NIRs and chemical analysis Fig. 4.

### Sample preparation for DNA extraction

Dried wood cores are drilled by Dremel 3000 to obtain 50–100 mg powder then placed in a new 1.5- or 2 mL tube. Clean the Dremel by using 70% Alcohol before continuing to the next sample. Next step, add 2 mm 3–5 beads and a bit of sterile sand into each tube and grind with TissueLyser II from Qiagen at 25 Hz for 5 min. The genomic DNA can be extracted using modified CTAB method [6]. The CTAB buffer contains 10% CTAB, Tris–HCl, 5 M NaCl, 0.5 M EDTA, 1% PVP, β-Mercaptoethanol, and dH2O.

### Sample preparation for anatomy analysis

Boil the wood core (only for hardwood) for approximately 30 min. Section the wood core at the cross, radial, and tangential directions using a sharp knife. Give a safranin, dehydrate the samples, and place on an object-glass then mount by using Entellan. Close with a cover glass to make a permanent slide. Observe the anatomical structure under the microscope [7].

### Sample preparation for Near- Infrared Spectroscopy (NIRs) analysis

Mix 3–5 individual cores as a composite sample. In total, 45 compositesamples were used in this study. Grind the wood core to obtain 900–1500 mg of wood powder in 40–60 mesh size [5]. Process the samples in the NIR Instrument.

### Sample preparation for chemicals analysis using LC-MS/MS

The samples may be used further for chemical analysis after the NIR. Weight the wood core as much as 500 mg . Samples are extracted with 5 ml of ethanol. Sonicate and filter the samples for 30 min with a 0.2-micrometer filter. Inject the sample into an instrument for liquid chromatography-tandem mass spectrometry (LC-MS/MS).
